# Reducing home infusion CLABSI through a dashboard and toolkit implementation

**DOI:** 10.1017/ice.2025.10385

**Published:** 2026-05

**Authors:** Susan Hannum, Jill Marsteller, Ayse P. Gurses, Sara E. Cosgrove, Ilya Shpitser, Helen Guo, Trung Phung, Opeyemi Oladapo-Shittu, Eili Y. Klein, Sara C. Keller, For The Home Infusion CLABSI Prevention Collaborative

**Affiliations:** 1 Department of Health Behavior and Society, Johns Hopkins Bloomberg School of Public Health, Baltimore, MD, USA; 2 Department of Health Policy and Management, Johns Hopkins Bloomberg School of Public Health, Baltimore, MD, USA; 3 Armstrong Institute of Patient Safety and Quality, Johns Hopkins Medicine, Baltimore, MD, USA; 4 Department of Anesthesiology and Critical Care, Johns Hopkins University School of Medicine, Baltimore, MD, USA; 5 Department of Emergency Medicine, Johns Hopkins University School of Medicine, Baltimore, MD, USA; 6 Division of Infectious Diseases, Department of Medicine, Johns Hopkins University School of Medicine, Baltimore, MD, USA; 7 Department of Hospital Epidemiology and Infection Control, Johns Hopkins Health System, Baltimore, MD, USA; 8 Department of Computer Science, Johns Hopkins Whiting School of Engineering, Baltimore, MD, USA

## Abstract

**Objectives:**

To evaluate the implementation and effectiveness of a novel home infusion central line-associated bloodstream infection (CLABSI) and home infusion-onset bloodstream infection (HiOB) dashboard and prevention toolkit.

**Design:**

Mixed methods study.

**Setting and Participants:**

Five home infusion agencies participating in the first CLABSI prevention collaborative.

**Methods:**

Agencies uploaded CLABSI and HiOB data to a comparative dashboard. The dashboard started in December 2022 and accepted retrospective data from June 2021. A CLABSI prevention toolkit was made available in June 2024. Using an interrupted time series, we present CLABSI and HiOB rates before and after dashboard and toolkit implementation. We surveyed and interviewed participants about the tools and toolkit, using directed content analysis to analyze the interviews.

**Results:**

After dashboard implementation, there was a decrease in CLABSI (−0.23/10,000 home-CVC days, 95% CI −0.28 to −0.18) and HiOB (−0.25/10,000 home-CVC days, 95% CI: −0.31 to −0.18) over time. With toolkit implementation, there was a further decrease in CLABSI (−0.17/10,000 home-CVC days, 95% CI: −0.30 to −0.044) and HiOB (−0.23/10,000 home-CVC days, 95% CI: −0.37 to −0.089) over time. Themes were associated with use of the tools (accessible, adaptable, patient-centered tools; user-friendly education to enhance understanding; barriers identified; tool mismatches; and strategies for tool delivery) and toolkit implementation (structural barriers, user-centered design, collaborative engagement and communication, toolkit used to enhance workforce competency, and concerns related to consistency).

**Conclusions:**

Implementation of a dashboard and a CLABSI prevention toolkit were each associated with both CLABSI and HiOB reduction in a collaborative of home infusion agencies.

## Introduction

As healthcare seeks to improve patient experience and reduce costs, central venous catheters (CVCs) are increasingly maintained in homes via home infusion therapy.^1^ Examples of home infusion therapies include parenteral chemotherapy,^2,3^ hydration, parenteral nutrition, outpatient parenteral antimicrobial therapy,^4^ intermittent infusions (eg, iron, factor, immunoglobulin, etc), and life-sustaining inotropic therapies. Over three million patients receive home infusion therapy annually in the United States.^1,5^ Patients with CVCs in homes are at risk of central line-associated bloodstream infections (CLABSI). Home infusion CLABSI have been estimated to occur at a rate of 0.2–0.24/1000 CVC-days, less than that reported in acute care hospitals.^6,7^ CLABSI in ambulatory settings have high morbidity and mortality, with 24% of patients presenting to hospitals with CLABSI admitted to an intensive care unit and 11% dying.^8^

Evidence-based guidelines for CLABSI prevention are well-established and routinely implemented in acute care,^9,10^ but the dramatically different environment of the home presents unique challenges. While many hospital-based prevention strategies may not translate effectively to homes due to these differences,^10,11^ one approach that has proven essential across healthcare settings is continuous monitoring of infection rates.^12,13^ No systematic framework exits for implementing such monitoring in home care, where definitions, surveillance methods, and data collection processes differ substantially from acute care.^6,14–17^ Consequently, it remains unclear whether monitoring strategies that have succeeded in hospitals can be adapted to reduce infections in home infusion.^6^ There is no national strategy to collect standardized data from home infusion agencies,^12^ and no national, or even multi-site, longitudinal home infusion CLABSI surveillance data using a validated CLABSI definition.

An additional fundamental difference between home infusion and acute care regarding CLABSI prevention is that patients and caregivers [rather than trained healthcare workers (HCWs)] carry out much of the day-to-day CVC maintenance activities. Patients are in a less controlled home environment (and often traveling outside the home environment for work, school, and other activities), are usually not critically ill, and often maintain their CVCs for months or years rather than days.^18^ Patients may have multiple caregivers and multiple healthcare locations (eg, clinics, infusion centers, etc.) accessing their CVCs. Although home infusion agencies have developed bundles of strategies to train patients and caregivers on CLABSI prevention, their efficacy has not been well evaluated.^15,19–21^

The Home Infusion CLABSI Prevention Collaborative (HICPC) focuses on CLABSI and home infusion-onset bloodstream infection (HiOB) surveillance and prevention practices. The HICPC validated and implemented home infusion CLABSI and HiOB definitions and is currently the only network of organizations in the country that benchmarks home infusion CLABSI using a validated definition.^22^ As the National Healthcare Safety Network (NHSN) does not have a module for home infusion,^12^ the HICPC is the first and so far only group of home infusion organizations in the United States to develop and implement a dashboard for CLABSI and HiOB reporting using validated surveillance definitions.^19,23^ It is also the first to disseminate tools for CLABSI prevention in the home, including videos, handouts, cognitive aids, and protocols. In this paper, our goals are to use multiple methods to: (1) determine the impact of the dashboard and prevention toolkit on CLABSI and HiOB rates over time, and (2) understand how the CLABSI prevention tools were implemented.

## Methods

### Setting and participants

The HICPC started in May 2019. HICPC members included five large home infusion agencies: three in the mid-Atlantic and two in the Midwest, covering portions of thirteen states and Washington, DC. Primary champions of the HICPC included two to six members of each infusion agency, who disseminated information to their agencies. The study was approved by the Johns Hopkins Institutional Review Board (protocol IRB00259916).

### Intervention

Learning collaborative activities included monthly webinars and a program listserv in which respondents discussed surveillance and prevention activities. In addition, HICPC members met with each other at professional conferences and visited each other’s agencies. HICPC members first underwent training in how to apply the CLABSI and HiOB definitions.^6^ In December 2022, using participatory design,^19^ we implemented a dashboard for HICPC members to retroactively upload CLABSI and HiOB data from June 2021.^6,19^ Collaborative members could compare their agency’s rates with other agencies and investigate subpopulations of interest (eg, pediatric patients, patients receiving chemotherapy, etc).

**Figure 1.**
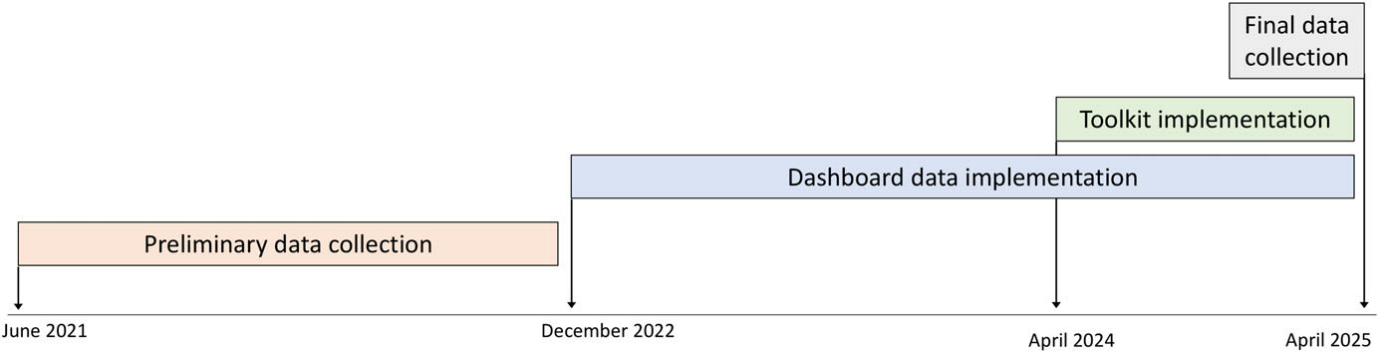
Timeline of study activities.

Using participatory design, HICPC members contributed to a toolkit for CLABSI prevention. Tools included those used to aid home infusion agency members in teaching patients and caregivers and intended for distribution to patients and caregivers such as handouts, cognitive aids, and videos (Table 1). The toolkit was implemented via the HICPC website (www.nhicpc.org) in June 2024. Patient education tools were approved and implemented per each agencies’ standard approval processes; thus, each agency chose which tools it wanted to use, modified non-video tools for their own use (eg, adding the agency name, changing images to reflect specific brands of equipment, etc), and implemented tools at different times (Figure 1). Several agencies had used versions of certain tools prior to toolkit implementation.

**Table 1.** Components of toolkit

Topic	Available via written document	Available via video
Home infusion overview for all home infusion patients and introduction to IV lines	x	X
Equipment considerations for general home infusion patients	X	
Bathing with an intravenous line		X
Intravenous push medications	X	X
Elastomeric device medications	X	X
Electronic pump medications	X	X
Taking down chemotherapy	X	X
Parenteral nutrition	X	X
Changing an inotropic medication bag	X	
Using flow regulator medications	X	X
Using gravity flow medications	X	X
Medication bag with vial	X	X
Flushing ports		X
Flushing the unused side of IV	X	X
Adding medication to elastomeric device	X	X
Drawing a medication from a vial or ampule	X	X
Lock therapy	X	
Chlorhexidine bathing	X	
Considerations for patients at high risk of CLABSI	X	
Evaluation of a patient who has experienced a CLABSI	X	
Saline-administer-saline or saline-administer-saline-heparin cognitive aid	X	
Nursing competency assessment	X	
Site care algorithm	X	
Patient-directed dressing bundle	X	
Hand hygiene	X	

### Semi-structured interviews

We conducted semi-structured interviews with home infusion nurses. We used a combination of purposive, convenience, and snowball sampling to recruit interview participants.^24^ We started by purposively recruiting staff members known to be actively engaged in CLABSI prevention activities and asked these individuals who else at their agency might be eligible. We also asked that HICPC champions disseminate a recruitment email to front-line nurses at their agencies. We interviewed at least two individuals from each agency.

Semi-structured interview guides explored how tools and the toolkit were used in CLABSI prevention. To ensure consistency, interviews were conducted by a single team member (SCK). After obtaining written consent, interviews were conducted via audio-recorded videoconference and lasted between 15 and 30 minutes. Interviews occurred between August 2024 and February 2025. We entered deidentified transcripts into MAXQDA for qualitative data management and analyses (VERBI Software, Berlin, GA).

### Surveys

We developed an electronic survey focused on tool implementation using the reach, effectiveness, adoption, implementation, and maintenance (RE-AIM) model (Appendix 1, Qualtrics XM, Seattle, WA).^25^ The survey included 29 multiple-choice and free-text items and took approximately 10 to 15 minutes to complete. Each agency was asked to electronically disseminate the survey to employees one month after implementation of the toolkit. Reminders were sent out every other week, three times. Responses were anonymous.

In addition, we developed a structural assessment focused on the makeup of the agency that we disseminated to one champion from each agency.

### Use of dashboard and toolkit

We recorded data from nhicpc.org, including access and viewing data (April 2024–April 2025) and tool-download data (June 2024–April 2025). This data reflected views and downloads by home infusion staff, typically by a home infusion agency for modification and local dispensing to other agency members and to patients and caregivers. As videos were available via QR code from toolkit documents, directly from nhicpc.org, and from YouTube.com (Alphabet, Mountain View, CA), we present aggregate numbers of video views reflective of views by patients.

### Impact of dashboard and toolkit on CLABSI and HiOB rates

An interrupted time series analysis (ITSA) to account for differences in rates over time was used to assess changes in CLABSI and HiOB rates (Stata Ver. 18, College Station, TX). CLABSI and HiOB were modeled separately. To assess the impact of the dashboard, pre-implementation rates were compared to post-implementation rates. Similarly, toolkit implementation was measured before and after the dissemination of the toolkit. The model for both CLABSI and HiOB included variables representing the pre-dashboard implementation trend, a step and slope change for the dashboard implementation, and a step and slope change for the toolkit implementation. Month 0 was the month from which data was first uploaded after validation and training in use of the definitions (June 2021).^6^

### Analysis of qualitative data

Deductive and inductive coding was used to analyze interviews.^26^ We first deductively applied a structured set of codes focused on tool use, approaches to training patients and caregivers as well as HCWs, positive attributes of the tools, changes made to the tools, and barriers, facilitators, and strategies for use of the tools. Two researchers applied these codes to three interview transcripts (SCK and SH) and then inductively coded 45 emergent sub-codes. Disagreements in coding were discussed throughout the codebook development phase and rectified to coder agreement.^26^ Thereafter, remaining transcripts and observation forms were single-coded using the established codebook. To verify code application and assess overall trustworthiness and congruence of interpretation, all code reports were reviewed (SCK and SH). Preliminary findings were presented to the research team for the opportunity to provide input on emergent findings and analyses.

### Analysis of survey data

For survey data, we reported frequencies and performed chi-squared, t-test, and ANOVA analyses as appropriate using Stata Ver. 18 (Stata, College Station, TX).

## Results

### Analysis of CLABSI and HiOB rates

**Figure 2.**
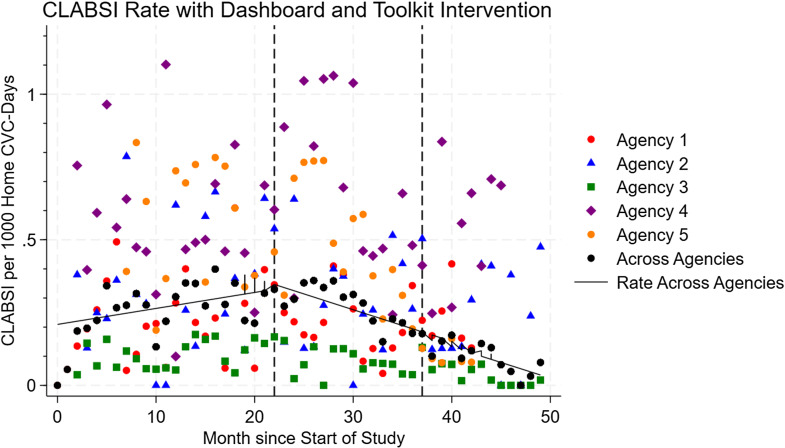
CLABSI over time, per month, per 1,000 home-CVC days. Vertical lines indicate implementation of the CLABSI monitoring dashboard and implementation of the CLABSI prevention toolkit.

CLABSI occurred at a mean rate of 2.2/10,000 home-CVC days (Figure 2, Table 2). After dashboard implementation, there was no immediate change in CLABSI (−0.13/10,000 home-CVC days, 95% CI: −0.86 to 0.61), but over time, there was a decrease in monthly CLABSI (−0.23/10,000 home-CVC days, 95% CI: −0.28 to −0.18). After toolkit implementation, there was an immediate decrease in CLABSI (−1.2/10,000 home-CVC days, 95% CI: −2.2 to −0.14) and a decrease in monthly CLABSI over time (−0.17/10,000 home-CVC days, 95% CI: −0.30 to −0.044).

**Table 2.** Changes in monthly CLABSI and HiOB rates after dashboard implementation and toolkit implementation, per 10,000 home-CVC days

	Immediate change after dashboard implementation (95% CI)	Change over time after dashboard implementation (95% CI)	Immediate change after toolkit implementation (95% CI)	Change over time after implementation (95% CI)
CLABSI	−0.13 (−0.86 to 0.61)	−0.23 (−0.28 to −0.18)	−1.2 (−2.2 to −0.14)	−0.17 (−0.30 to −0.044)
HiOB	0.14 (−0.73 to 1.0)	−0.25 (−0.31 to −0.18)	−1.0 (−2.2 to 0.078)	−0.23 (−0.37 to −0.089)

**Figure 3.**
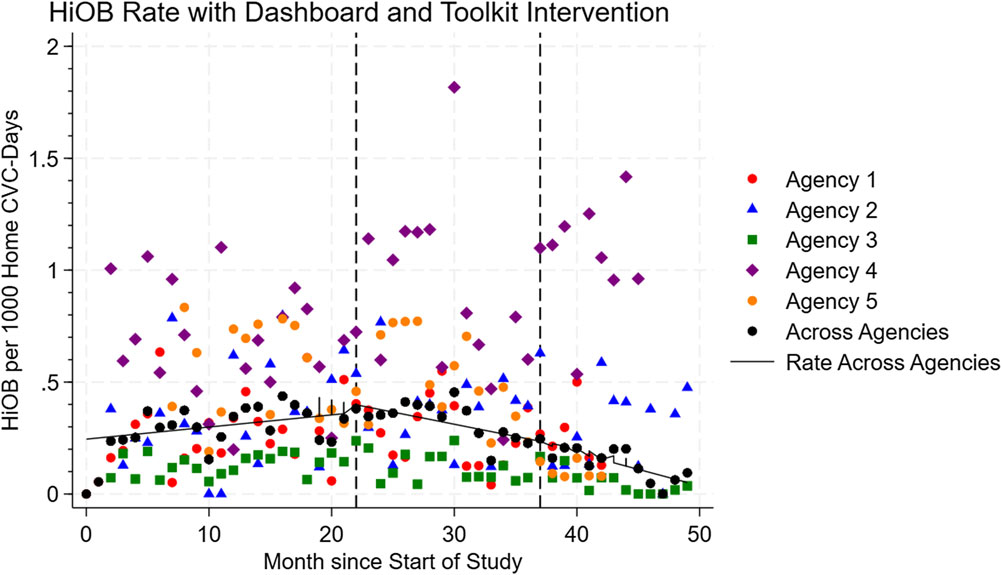
HiOB over time, per month, per 1,000 home-CVC days. Vertical lines indicate implementation of the CLABSI monitoring dashboard and implementation of the CLABSI prevention toolkit.

HiOB occurred at a mean rate of 2.6/10,000 home-CVC days (Figure 3). After dashboard implementation, there was no immediate change in HiOB (0.14/10,000 home-CVC days, 95% CI: −0.73 to 1.0), but there was a decrease in monthly HiOB over time (−0.25/10,000 home-CVC days, 95% CI: −0.31 to −0.18). After toolkit implementation, there was no immediate change in HiOB (−1.0/10,000 home-CVC days, 95% CI: −2.2 to 0.078), but there was a decrease in monthly HiOB over time (−0.23/10,000 home-CVC days, 95% CI: −0.37 to −0.089).

Over 13 months, the website was viewed 4218 times, the dashboard was viewed 1217 times, and tools were downloaded 345 times (Appendix 2). Videos were viewed 82,601 times (data not shown).

### Description of survey data

Agencies ranged in size from 75 to 1500 employees (Appendix 3). All agencies employed their own home health or home infusion nurses and worked with affiliated home health or home infusion nurses. Of 26 respondents to a survey about the toolkit (Appendix 4), 88% strongly or somewhat agreed with the statement, “I like these tools” (Appendix 5).

### Interviews

We interviewed 17 participants about their use of the tools (Table 3) and the toolkit (Table 4). We describe five primary emergent themes regarding the uptake of individual tools and five themes regarding the uptake of the overall toolkit.

**Table 3.** Themes and items of importance related to how individual tools are used

Theme	Items of importance	Example quote
***Accessible, adaptable, patient-centered tools support ongoing patient use***	• Participants used internet-based videos to supplement in-person training • Participants used printed materials in teaching patients • Participants adapted tools to patient needs and learning styles • Participants used tools to provide personalized, tailored information for each patient • Participants focused on ensuring consistency in messaging • Participants gamified instructions and used slogans • Participants made tools easy to access	“Patients can lay [the tool] out, follow it step by step, and it just details the steps that should be happening, you know, how to flush to saline, how to, you know, if you meet resistance, call in, it has a lot of information but it’s an easy flow to it so they can just follow along.” Agency 4, Nurse “There’s … all sorts of other educational tools on there that are wonderful, given … what you want to focus on with that patient, are they really nervous about bathing with their line, there’s specific information about that, or are they worried about infection, and then there’s specific information about that. So you can kind of tailor it to what each patient needs.” Agency 3, Infection Preventionist
***User-friendly educational tools enhance patient understanding***	• Participants appreciated that QR codes linking to online videos provided access to multiple tools • Participants appreciated that tools can be easily updated and adapted	“And so the idea would be to link it so that people can get to it quickly and it becomes a thing that people know. It’s a resource that exists if I push this button.” Agency 5, Nurse Infection Preventionist
***Barriers to understanding and implementing tools***	• Literacy and numeracy challenges impacted patient understanding • Patients who felt overwhelmed or anxious could not absorb information • Variable home environments made it difficult to implement all tools • Physical dexterity limitations made some tasks difficult for patients • Some participants noted difficulty assessing patient understanding	“Anyone who’s got tremors or who has weakness in their hands has a harder time with [performing infusions], and we need a tight connection to make sure things don’t leak.” Agency 5, Nurse Infection Preventionist “I mean [family members] will YouTube everything and everything, so we tell them to be careful of that because every institution does things a little bit differently” Agency 3, Nurse
***Tool mismatches with patient needs***	• Tools can become outdated and require revisions • Some tools were considered too lengthy • Some tools did not match agency equipment specifications	“My only … concern … is that how quickly these videos might become outdated based on standards changing or equipment changing within the videos that could cause confusion for the patients later on down the line, but that is not a current issue now.” Agency 3, Nurse
***Strategies for improving access and delivery of educational tools and resources***	• Some agencies arranged tool distribution upon staff arrival • Some agencies maintained a database for staff to access tools • Some agencies provided printed materials for immediate use • Some agencies shared information during staff meetings • Some agencies used online training modules • Some agencies used patient portals	“Yeah, it’s typically printed-out documents. We have like our core kind of component that is printed out. It lives in the document so we can modify it as we need to and then we’re able to print it out. And the other pieces are added to it…. It’s all printed materials.” Agency 3, Nurse

**Table 4.** Qualitative analysis of toolkit implementation

Theme	Items of importance	Example quote
***Structural barriers to implementation***	• Inadequate buy-in • Lack of platform to disseminate information to patients or HCWs • Lack of internet in homes • Lack of awareness of tools • Competing priorities • Staffing shortages • Cost of some tools • Technological literacy	“I think there’s going to be some challenge getting buy-in … What I’m hoping is we get a cohort of patients that really find it great, and then we can just kind of be like, see, these guys did it … yay, and the other nurses will kind of go, oh, that’s really cool.” Agency 5, Nurse “We are within our Joint Commission window for survey. So, of course there’s so much focus that is pinned on Joint Commission and compliance with that also, that that’s the reason that the [toolkit], unfortunately, is sort of on the back burner until those things are in better order and completed.” Agency 1, Nurse Manager
***User-centered design as facilitators of adoption***	• Visual nature of tools easier for patients to use • Familiar tools are easier to adapt • New workflow (including using technology) facilitates disseminating information • Easy to access	“[Patients prefer] a diagram or an actual picture of the objects that you’re using — specifically flushes, alcohol…. I know myself if you give me something to read, I can read it, of course I can. But I actually learn better by looking at a diagram or physically doing it.” Agency 4 Nurse “All of our documentation now is all on their phone. …. And so, having that phone almost in – and I hate to say in the patient’s face – but in the front of the patient and on is gonna be even more apt for them to have it on and flip that in front of them to show them a video and be more: ‘Hey, let me just throw this in front of you. Let me show you this video.’” Agency 2 Nurse Manager
***Collaborative engagement and communication to enhance implementation***	• Learn from others in the collaborative • First implement toolkit with the highest-risk, highest-impact groups • Use the tools to teach healthcare workers • Reorganize workflows to change who disseminates the tools • Use meetings and emails for staff and patient portal for patients • Allow others to take credit	“Well, we decided to implement that with our new TPN patients, the new starts, with our agency and home health side providing the care.” Agency 1 Nurse “Sometimes [nurses] have to think it’s their own idea, do you know what I mean? Like I can tell them all day long, like these are so cool, you should use them, and they’re like, nah, nah, and then they find them and they’re like, oh, these are really cool.” Agency 5 Nurse Manager
***Enhancing workforce competency via consistent, engaging training***	• Encourages initiatives • Uses to onboard nurses • Ensure consistency	“One of the goals that we have here is … have those educational videos be available not only to our patients, but to the nurses and the agencies that we work with. So that if an agency has a new nurse that comes on, we would want them to watch the video on how to teach their patient to properly shower when they have an IV access. How to properly scrub the hub.” Agency 1 Nurse “Maybe a resident or someone comes in and says, ‘Oh, you’re going to be at home, you’re going to have a nurse in there all the time, they’re going to do it for you’, and then the patient gets home thinking we’re coming every day, so having the videos where it kind of explains what to expect.” Agency 4 Nurse
***Challenges and concerns related to consistency, patient engagement, and usability***	• Concerns about different practices or equipment • Concerns that patients will skip instructions • Length of tools may impede use	“I could see confusion if the supplies are different than what is being supplied to the patient and it may cause patient confusion.” Agency 4 Nurse “It’s also lengthy. It’s several pages long, and that is something we’re looking at hopefully being able to revise with some of these tools, because we know patients don’t read all of that information.” Agency 5 Nurse

### Themes involving use of tools

Respondents noted that accessible, adaptable, patient-centered tools supported ongoing patient use of the tools. User-friendly educational tools could enhance patient understanding. However, there were barriers to understanding and implementing the tools, such as literacy and numeracy challenges, feeling overwhelmed or anxious, variable home environments, physical dexterity, and difficulty assessing patient understanding. At times, the tools did not match what patients needed. Respondents used agency resources for improving access to and delivery of educational tools and resources.

### Themes involving toolkit implementation

We noted five themes focused on toolkit implementation. There were structural and resource barriers to implementation. Insufficient buy-in from leadership and frontline staff posed a significant challenge. Additionally, agencies that lacked effective dissemination platforms struggled to provide information to patients and HCWs. Access issues—including lack of internet connectivity in patients’ homes and low levels of technological literacy—restricted engagement with digital tools. Competing demands and staffing shortages diminished capacity to support new interventions. As a second theme, user-centered design facilitated toolkit adoption. As a third theme, collaborative engagement and communication, along with strategic implementation and allowing others to take credit, also enhanced implementation. As a fourth theme, some respondents noted that the toolkit itself improved workforce competency. Some agencies even used the toolkit to onboard nurses. Meanwhile, using the toolkit ensured consistency in care. Finally, the overall dissemination of the toolkit required overcoming concerns. Concerns about variability in practices or equipment between what is shown on a video and what an agency may use may hinder implementation. Additionally, there was apprehension that patients might not follow complicated instructions.

## Discussion

For the first time, we investigated the impact of a CLABSI dashboard and toolkit on CLABSI and HiOB in a collaborative of home infusion therapy agencies. The dashboard was associated with a decrease in both CLABSI and HiOB over time. In the presence of the dashboard, the toolkit was associated with an immediate decrease in CLABSI as well as a decrease in both CLABSI and HiOB over time. Ultimately, the efforts of the HICPC resulted in CLABSI and HiOB ending at half the rate of the initial measurement.

Access to longitudinal data on healthcare-associated infection (HAI) in comparison with similar organizations is an important driver of HAI prevention.^27–30^ Inclusion of acute care CLABSI data in NHSN benchmarking has been associated with a reduction in acute care CLABSI.^31^ We showed that dashboard implementation also can reduce home infusion CLABSI and HiOB. Other home infusion agencies implementing a CLABSI surveillance dashboard may consider similar methods, with an analysis plan that could include an approach such as statistical process control.

Meanwhile, CLABSI prevention bundles have successfully reduced CLABSI in acute care hospitals and are recommended by national guidelines.^10^ In this case, a toolkit for home infusion CLABSI prevention based on acute care guidelines^10^ was associated with a decrease in CLABSI and HiOB. While the evidence base for CLABSI prevention approaches in homes, where patients and caregivers perform most of the care and where the environment is vastly different from a hospital, needs to be expanded, we have shown that these tools can lead to a reduction in home infusion CLABSI. However, not all agencies fully implemented all components of the toolkit. Understanding which components of the home infusion CLABSI prevention toolkit were most impactful would be important in triaging resources.

Most were satisfied with the tools and thought they were useful for patients, particularly videos and cognitive aids. However, some were concerned that images of equipment may lead to confusion if these were different than what the agencies used. For example, a video may use a different brand of an infusion pump than the agency uses. Those developing tools for CLABSI prevention in home care settings should balance variation with standardization.^32^

Overall use of the toolkit was high. Videos were viewed 82,601 times. It is unlikely that HICPC staff or patients alone viewed the videos this frequently. It is possible that members of the public found the videos through internet searches. We advise that experts in infection prevention examine instructional tools available to the public to ensure that patients are receiving accurate information.

In prior work, we developed HiOB as a metric that would be easier to measure than home infusion CLABSI and showed that this metric closely approximates CLABSI.^6^ We have now showed that a dashboard and a toolkit associated with reduction in home infusion CLABSI were also associated with reduction in HiOB. As acute care hospitals implement healthcare-onset bloodstream infection (HOB) surveillance,^33–35^ understanding whether interventions geared toward reducing acute care CLABSI also reduce HOB will be important.

There was variation in CLABSI and HiOB rates by agency. While determining the specific facilitators or strategies that could have led to lower CLABSI and HiOB rates in particular agencies was out of the scope of this study, a qualitative analysis focused on a positive deviance methodology^36^ would be helpful. In addition, high-performing agencies could present their strategies to other agencies.

The work described here occurred during and after the COVID-19 pandemic. In the first month of the data collection (June 2021), no CLABSI or HiOB were reported. While this may have been an outlier, the general trend pre-intervention was an increase in CLABSI and HiOB through December 2022. In acute care hospitals, the pandemic was associated with an increase in CLABSI through 2021 that then declined in 2022,^37,38^ when CLABSI and HiOB rates in our study were still increasing. Home infusion CLABSI and HiOB rates did not decline until after the December 2022 dashboard implementation. As there are no other published or publicly available longitudinal home infusion CLABSI rates for comparison, we do not know how COVID-19 impacted home infusion CLABSI. However, some challenges noted such as competing priorities likely were even more of a challenge during COVID-19 due to staffing shortages and turnover. Engagement of leadership up front and through in-person visits increased engagement in the project.

Our study had several limitations. First, home infusion agencies implemented different portions of the toolkit at different times. Second, some agencies did not implement the entire toolkit due to other priorities, staffing shortages, or lack of a platform to disseminate information. Despite this, there was a decrease in CLABSI and HiOB rates after toolkit implementation. Third, the collaborative included five home infusion agencies that were all affiliated with academic medical centers. Results may have differed if the dashboard or toolkit were implemented more widely. Fourth, low numbers of participants responded to the survey or participated in interviews. We did achieve thematic saturation in the interview data.

Overall, in this first large-scale study of CLABSI prevention approaches in home infusion therapy, we showed that a comprehensive process for monitoring, surveillance, and benchmarking used for CLABSI and HiOB reporting was associated with a decrease in CLABSI and HiOB in home infusion agencies over time, and an added toolkit for home infusion CLABSI prevention was associated with an immediate further decrease in CLABSI and a decrease in both CLABSI and HiOB over time. This suggests that approaches and tools adapted from acute care settings for the home care setting, with input from home infusion professionals, can be successful in home infusion CLABSI and HiOB prevention. Many outside the collaborative likely used the tools especially the videos, pointing to the broader need for evidence-based reliable tools for home infusion CLABSI prevention. In future iterations of the toolkit, we may consider focusing on videos and on making written materials more adaptable, while still providing a menu of options for home infusion agencies. A home infusion CLABSI dashboard and toolkit should be disseminated more widely.

## Supporting information

Hannum et al. supplementary material 1 Hannum et al. supplementary material

Hannum et al. supplementary material 2 Hannum et al. supplementary material

Hannum et al. supplementary material 3 Hannum et al. supplementary material

Hannum et al. supplementary material 4 Hannum et al. supplementary material

Hannum et al. supplementary material 5 Hannum et al. supplementary material

## Supplementary material

The supplementary material for this article can be found at https://doi.org/10.1017/ice.2025.10385.
