# Supplementary material for: Reducing home infusion CLABSI through a dashboard and toolkit implementation
Source: Infect Control Hosp Epidemiol. 2026 Jan 21;47(5):433–40. doi: 10.1017/ice.2025.10385 (PMC12885047; doi:10.1017/ice.2025.10385)
Supplement: Hannum et al. supplementary material 1 — Hannum et al. supplementary material [file S0899823X25103851sup001.docx]

Appendix 1: Survey on Toolkit

1. How do you learn about new tools or tricks for teaching patients and caregivers? Pick all that apply.

--Conference

--Emails

--In-service or staff meeting

--Printed information (for example, posters, flyers, forms, etc.)

--Publicly available tools

--Internal electronic tools

--Other __________________________________________________

2. Do you use a checklist to help you assess patient or caregiver competency?

--No

--Yes

3. What do you focus on when assessing patient or caregiver competency? Pick all that apply.

--Bathing

--CVC care

--Dressing changes

--Hand washing

--Medication preparation

--Understanding the purpose of the treatment

--Understanding the need to care for the IV line

--Other __________________________________________________

4. How frequently do you reassess competency in a patient/caregiver who is deemed competent?

--Once a week

--Once a month

--Once a quarter

--Once a year

--Every 1-5 years

--Other __________________________________________________

Q8 Have you used any new CLABSI prevention or IV line maintenance tools within the last six (6) months?

- No (1)
- Yes (2)

Display this question:

If Have you used any new CLABSI prevention or IV line maintenance tools within the last six (6) months? = Yes

Q9 If yes, when did you start using the new tools? (Please provide an approximate date in the format MM/DD/YYYY)

________________________________________________________________

Display this question:

If Have you used any new CLABSI prevention or IV line maintenance tools within the last six (6) months? = Yes

Q10 If yes, how did you hear about the tools? Pick all that apply.

- Conference (1)
- Emails (4)
- In-service or staff training (9)
- Printed information (for example, posters, flyers, forms, etc.) (5)
- Publicly available tools (10)
- Internal electronic tools (7)
- Other (8) __________________________________________________

Q11 How frequently do you use each of the following tools?

|  | Never (1) | Rarely (2) | Sometimes (3) | Often (4) | Always (5) |
| --- | --- | --- | --- | --- | --- |
| Competency assessment form (10) |  |  |  |  |  |
| Bathing education tools (13) |  |  |  |  |  |
| Hand hygiene tools (14) |  |  |  |  |  |
| Instruction forms (15) |  |  |  |  |  |
| SAS or SASH mat (16) |  |  |  |  |  |
| Videos (17) |  |  |  |  |  |

Q12 How satisfied are you with each of the following tools?

|  | Extremely dissatisfied (11) | Somewhat dissatisfied (12) | Neither satisfied nor dissatisfied (13) | Somewhat satisfied (14) | Extremely satisfied (15) |
| --- | --- | --- | --- | --- | --- |
| Competency assessment form (11) |  |  |  |  |  |
| Bathing education tools (15) |  |  |  |  |  |
| Hand hygiene tools (16) |  |  |  |  |  |
| Instruction forms (17) |  |  |  |  |  |
| Patient education video (18) |  |  |  |  |  |
| SAS or SASH mat (19) |  |  |  |  |  |

Q13 Please rate the following on a scale of:

|  | Strongly disagree (11) | Somewhat disagree (12) | Neither agree nor disagree (13) | Somewhat agree (14) | Strongly agree (15) |
| --- | --- | --- | --- | --- | --- |
| These tools meet my approval. (1) |  |  |  |  |  |
| These tools are appealing to me. (9) |  |  |  |  |  |
| I like these tools. (10) |  |  |  |  |  |
| I welcome the use of these tools. (11) |  |  |  |  |  |
| These tools seem fitting. (12) |  |  |  |  |  |
| These tools seem suitable. (13) |  |  |  |  |  |
| These tools seem applicable. (14) |  |  |  |  |  |
| These tools seem like a good match. (15) |  |  |  |  |  |

Q14 One year from now, how likely is it that you will still be using the tools?

- Extremely unlikely (14)
- Somewhat unlikely (15)
- Neither likely nor unlikely (16)
- Somewhat likely (17)
- Extremely likely (18)

End of Block: Competency Assessment

Start of Block: Experience in Home Infusion therapy

Q15
**This last set of questions will ask you about your experience in Home Infusion therapy and some demography:**

Q16 Have you ever participated in a root cause analysis regarding a CLABSI?

- No (1)
- Yes (2)

Q17 Are you aware of your organization’s CLABSI rate?

- No (1)
- Yes (2)

Display this question:

If Are you aware of your organization’s CLABSI rate? = Yes

Q18 If yes, how did you find out? Pick all that apply.

- Emails (7)
- Posters/Flyers (8)
- Staff meeting (9)
- Website or dashboard (10)
- Other (11) __________________________________________________

Q19 What is your role in your organization? Pick all that apply.

- Administrator (1)
- Home Care Coordinator (2)
- Manager (3)
- Nurse (4)
- Pharmacist (5)
- Pharmacy Technician (6)
- Prefer not to say (8)
- Other (If other, specify in the text box below) (7) __________________________________________________

Q20 How many years have you been working at this organization?

________________________________________________________________

Q21 How many years have you been working in home care?

________________________________________________________________

Q22 What is your age in years?

________________________________________________________________

Q23 How would you describe your race/ethnicity? Pick all that apply.

- American Indian or Alaska Native (1)
- Asian (2)
- Black or African American (3)
- Native Hawaiian or Other Pacific Islander (4)
- White (5)
- Other (If other, specify in the text box below) (6) __________________________________________________
- Prefer not to say (7)

Q24 How would you describe your gender?

- Male (1)
- Female (2)
- Non-binary / third gender (3)
- Other (If other, specify in the text box below) (5) __________________________________________________
- Prefer not to say (4)

Q25 Any final comments on the tools?

________________________________________________________________

End of Block: Experience in Home Infusion therapy
