# Supplementary material for: Reducing home infusion CLABSI through a dashboard and toolkit implementation
Source: Infect Control Hosp Epidemiol. 2026 Jan 21;47(5):433–40. doi: 10.1017/ice.2025.10385 (PMC12885047; doi:10.1017/ice.2025.10385)
Supplement: Hannum et al. supplementary material 2 — Hannum et al. supplementary material [file S0899823X25103851sup002.pptx]

## Slide 1
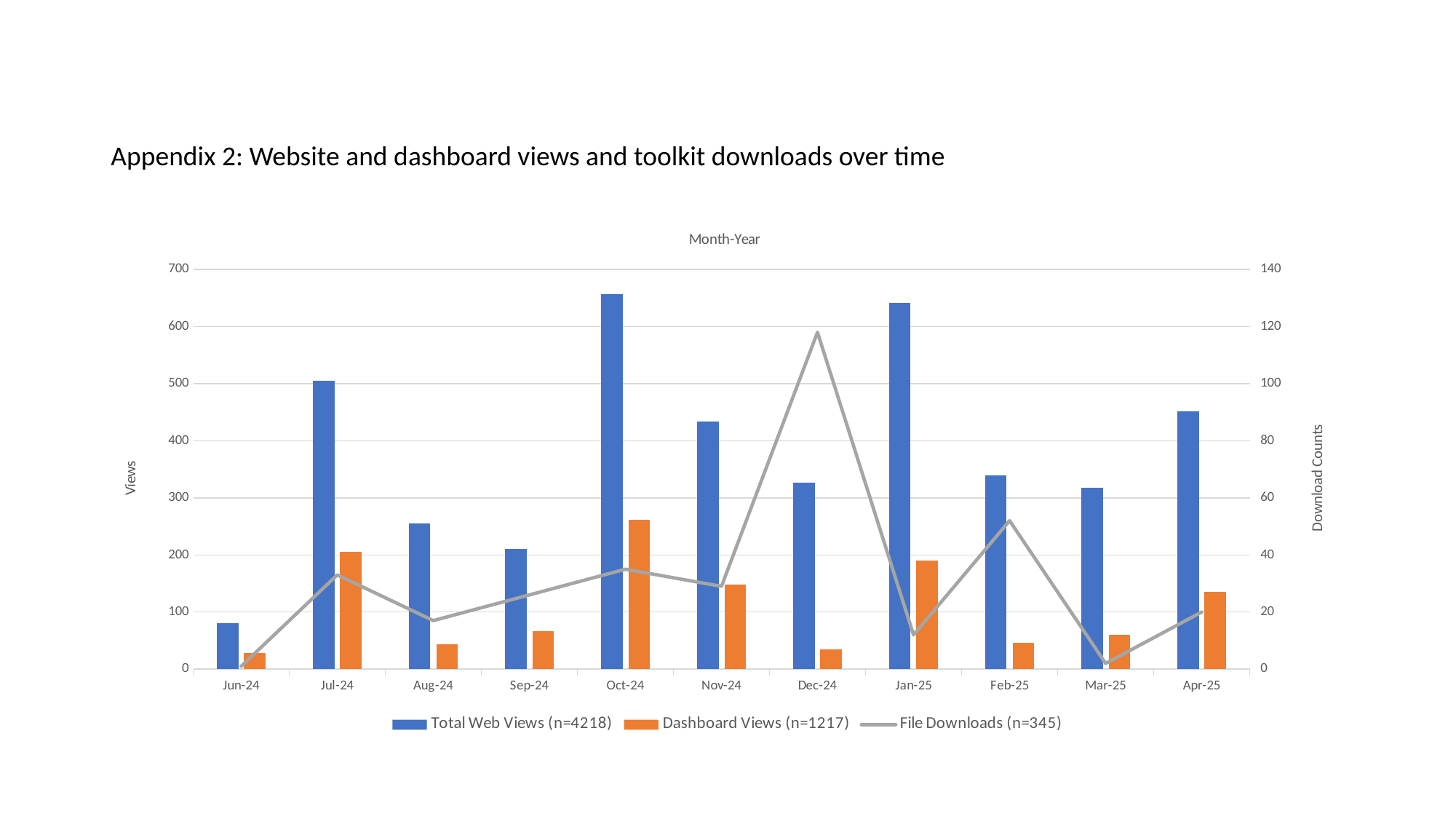

# Appendix 2: Website and dashboard views and toolkit downloads over time
### Chart
| Category | Total Web Views (n=4218) | Dashboard Views (n=1217) | File Downloads (n=345) |
|---|---|---|---|
| 45444 | 81.0 | 28.0 | 1.0 |
| 45474 | 505.0 | 205.0 | 33.0 |
| 45505 | 255.0 | 43.0 | 17.0 |
| 45536 | 210.0 | 66.0 | 26.0 |
| 45566 | 657.0 | 262.0 | 35.0 |
| 45597 | 434.0 | 148.0 | 29.0 |
| 45627 | 326.0 | 34.0 | 118.0 |
| 45658 | 641.0 | 190.0 | 12.0 |
| 45689 | 339.0 | 46.0 | 52.0 |
| 45717 | 318.0 | 60.0 | 2.0 |
| 45748 | 452.0 | 135.0 | 20.0 |
