# Supplementary material for: Reducing home infusion CLABSI through a dashboard and toolkit implementation
Source: Infect Control Hosp Epidemiol. 2026 Jan 21;47(5):433–40. doi: 10.1017/ice.2025.10385 (PMC12885047; doi:10.1017/ice.2025.10385)
Supplement: Hannum et al. supplementary material 3 — Hannum et al. supplementary material [file S0899823X25103851sup003.docx]

Appendix 3: Description of Participating Agencies and Tools Implemented

|  | Agency 1 | Agency 2 | Agency 3 | Agency 4 | Agency 5 |
| --- | --- | --- | --- | --- | --- |
| Number of employees | 250 | 394 | 1500 | 75 | 220 |
| Employ their own home health or home infusion nurses | Yes | Yes | Yes | Yes | Yes |
| Agency works with unaffiliated home health or home infusion nurses | Yes | Yes | No | Yes | Yes |
| Use nursing competency assessment | Yes | No | No | Yes | Yes |
| Use patient education sheets | Yes | Yes | Yes | Yes | Yes |
| Use site care algorithm | Yes | No | No | No | Yes |
| Use patient-directed dressing bundle | No | Yes | No | No | Yes |
| Use saline-administer-saline or saline-administer-saline-heparin mat | No | Yes | Yes | Yes | Yes |
| Use chlorhexidine baths for selected patients | No | No | Yes | No | No |
| Use videos | No | No | Yes | No | Yes |
