# Supplementary material for: Reducing home infusion CLABSI through a dashboard and toolkit implementation
Source: Infect Control Hosp Epidemiol. 2026 Jan 21;47(5):433–40. doi: 10.1017/ice.2025.10385 (PMC12885047; doi:10.1017/ice.2025.10385)
Supplement: Hannum et al. supplementary material 4 — Hannum et al. supplementary material [file S0899823X25103851sup004.docx]

| Characteristics | Survey participants,  n=26 (%) | Interview participants,  n=17 (%) |
| --- | --- | --- |
| **Age**: Mean (standard deviation) (missing=1) | 47.6 (8.3) | 47.0 (8.6) |
| **Gender**: Female | 23 (88.5) | 15 (88.2) |
| Male | 2 (7.7) | 2 (11.8) |
| Prefer not to say | 1 (3.9) | 0 (0) |
| **Race**: White | 23 (88.5) | 16 (94.1) |
| Native Hawaiian or Pacific Islander | 1 (3.9) | 1 (5.9) |
| Prefer not to say | 2 (7.7) | 0 (0) |
| **Role**: Educator | 2 (7.7) | 2 (11.8) |
| Home care coordinator | 3 (11.5) | 1 (5.9) |
| Infusion nurse | 14 (53.8) | 6 (35.3) |
| Manager/Director | 5 (19.2) | 5 (29.4) |
| Quality improvement or infection control | 2 (7.7) | 3 (17.6) |
| **Years in current role**: 0-4 | 6 (23.1) | 10 (58.8) |
| 5 - 9 | 7 (26.9) | 3 (17.7) |
| 10-14 | 5 (19.2) | 1 (5.9) |
| 15-19 | 6 (23.1) | 1 (5.9) |
| >20 | 2 (7.7) | 2 (11.8) |
| **Duration working in home care**: 0-4 | 5 (19.2) | 6 (35.3) |
| 5 - 9 | 10 (38.5) | 7 (41.2) |
| 10-14 | 3 (11.5) | 2 (11.8) |
| 15-19 | 3 (11.5) | 0 (0) |
| >20 | 3 (11.5) | 2 (11.8) |
| Unknown | 2 (7.7) | 0 (0) |

Appendix 4: Characteristics of home infusion professionals participating in survey and interviews.
