# Supplementary material for: Reducing home infusion CLABSI through a dashboard and toolkit implementation
Source: Infect Control Hosp Epidemiol. 2026 Jan 21;47(5):433–40. doi: 10.1017/ice.2025.10385 (PMC12885047; doi:10.1017/ice.2025.10385)
Supplement: Hannum et al. supplementary material 5 — Hannum et al. supplementary material [file S0899823X25103851sup005.docx]

Appendix 5: Respondents to survey about toolkit, of 26 respondents.

|  | Always or often use tool N= (%) | Extremely or somewhat satisfied with tool N= (%) | Strongly or somewhat agree with statement N= (%) |
| --- | --- | --- | --- |
| Bathing education tools | 11 (42.3) | 14 (53.8) | -- |
| Competency assessment | 7 (26.9) | 14 (52.8) | -- |
| Hand hygiene tools | 24 (92.3) | 22 (84.6) | -- |
| Instruction forms | 23 (88.5) | 24 (92.3) | -- |
| SAS or SASH mat | 21 (80.8) | 13 (50.0) | -- |
| Videos | 7 (26.9) | 22 (84.6) | -- |
| I like these tools. | -- | -- | 23 (88.5) |
| I welcome the use of these tools. | -- | -- | 22 (84.6) |
| These tools are appealing to me. | -- | -- | 22 (84.6) |
| These tools meet my approval. | -- | -- | 22 (84.6) |
| These tools seem applicable. | -- | -- | 23 (88.5) |
| These tools seem fitting. | -- | -- | 22 (84.6) |
| These tools seem like a good match. | -- | -- | 21 (80.8) |
| These tools seem suitable. | -- | -- | 21 (80.8) |
